# Polymorphisms of the *LTA* Gene May Contribute to the Risk of Myocardial Infarction: A Meta-Analysis

**DOI:** 10.1371/journal.pone.0092272

**Published:** 2014-03-18

**Authors:** Na Li, Runmei Liu, Hongxia Zhai, Liang Li, Yaxin Yin, Jinjin Zhang, Yunfeng Xia

**Affiliations:** First Cadres Ward, The First Affiliated Hospital of PLA General Hospital, Beijing, China; Scuola Superiore Sant'Anna, Italy

## Abstract

**Objective:**

The lymphotoxin-α (LTA), as one of the mediators of inflammation, may play an important role in the pathogenesis of myocardial infarction (MI). Genetic association studies (GAS) that have investigated the association between three common polymorphisms (A252G, G10A and C804A) of the *LTA* gene and susceptibility to MI have produced contradictory and inconclusive results. The aim of this meta-analysis is to provide a relatively comprehensive account of the association of these polymorphisms with susceptibility to MI.

**Methods:**

A literature search for eligible GAS published before October 15, 2013 was conducted in the PubMed, Embase, Web of Science, Cochrane Library, and CNKI (China National Knowledge Infrastructure) databases. We performed a meta-analysis of fifteen case-control studies with a total of 22,549 MI patients and 16,105 healthy controls.

**Results:**

For *LTA* A252G, a borderline significant overall association was found, indicating that GG genotype may confer an increased susceptibility to MI compared to AA and AG genotypes. Based on an ethnicity stratification analysis, a significant association was observed in Asians, but not in Caucasians. For *LTA* G10A, no significant overall association was found. However, subgroup analysis based on ethnicity suggested that the 10A allele may confer a significant increased susceptibility to MI only in Asian populations. For *LTA* C804A, the combined results revealed a significantly increased susceptibility to MI for carriers of the 804A allele in both overall analysis and stratified analyses.

**Conclusion:**

This meta-analysis shows that *LTA* C804A may be associated with an increased susceptibility to MI, whereas *LTA* A252G and G10A may confer a significant increased susceptibility to MI only in Asians. Thus, these polymorphisms of the *LTA* gene can probably be used with other genetic markers together to identify individuals at high susceptibility to MI especially in Asians.

## Introduction

Myocardial infarction (MI) remains the principal cause of death in many countries despite improvements in lifestyle and the development of new pharmacologic approaches [1]. According to data from NHANES (National Health and Nutrition Examination Survey) 2003 to 2006 the overall prevalence of MI is 3.6% in US adults over the age of 20, with rates of 4.7% for men and 2.6% for women [2]. The estimated average number of years of life lost due to MI is 15 [3]. MI, which is widely accepted as a chronic inflammatory disease, usually results from the rupture of atherosclerotic plaque with thrombus formation and the occlusion of the coronary vessel, resulting in an acute reduction of blood supply to a portion of the myocardium [4]. Plaque rupture with thrombosis is well established as a critical factor in the pathogenesis of MI [5]. Inflammatory mediators such as cytokines are involved in atheroma formation and rapid evolution of the atheromatous injury, leading to plaque rupture and MI [6]. Furthermore, epidemiological studies have revealed that MI is a complex multifactorial disease and is clearly influenced by environmental factors and genetic predisposition [7]–[9]. Functionally relevant polymorphisms in genes involved in the inflammatory pathways may cause acute thrombus formation over the plaque with abrupt vessel closure and affect an individual's susceptibility to MI [10].

**Figure 1:**
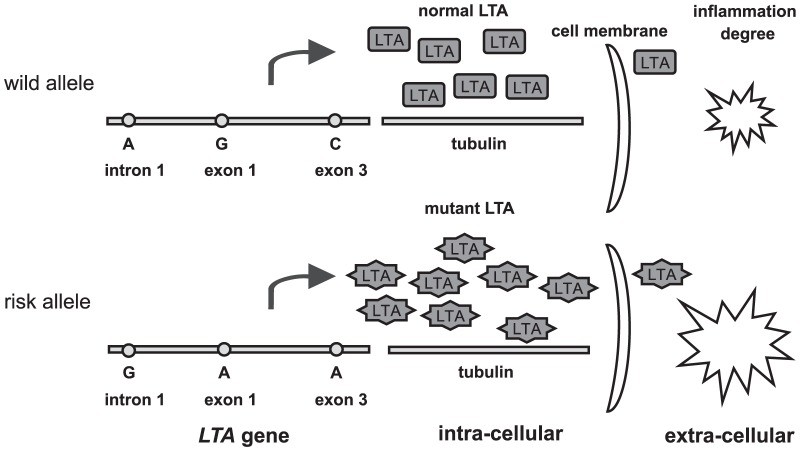
The potential roles of SNPs in the *LTA* gene in inflammatory process and the pathogenesis of myocardial infarction.

Lymphotoxin-α (LTA) is one of the cytokines produced in the early stages of vascular inflammatory processes [11]. LTA has been implicated in the pathogenesis of atherosclerosis and coronary heart disease (CHD) [12]. Since LTA is an inflammatory mediator, it is likely that functional variations in the gene encoding this protein confer a high susceptibility to MI by affecting the degree of inflammation at the lesion, as shown in Figure 1. Normal LTA protein, trafficking by binding to intra-cellular tubulins, can induce adhesion molecules and cytokines from vascular endothelial cells, vascular smooth-muscle cells, and several kinds of leukocytes, thereby contributing to the inflammatory process [13]. However, these inflammatory biological activities could be influenced by amino-acid substitutions in LTA, such as A-to-C in intron 1, G-to-A in exon 1, and C-to-A in exon 3. Thus, it seems that increased level of functionally mutant LTA protein is associated with increased degree of inflammation, thereby conferring a higher susceptibility to MI.

The *LTA* gene, which encodes LTA on chromosome 6p21, has been linked with the risk of MI [14]. The single nucleotide polymorphisms (SNPs) in the *LTA* gene seem to be involved in inflammation by both qualitatively and quantitatively modifying the function of the LTA protein [15]. In this respect, many researchers have tested three common polymorphisms in the *LTA* gene for genetic association with MI risk, including A252G (dbSNP: rs909253) in intron 1, G10A (dbSNP: rs1800683) in exon 1 and C804A (dbSNP: rs1041981) in exon 3. Their results, however, have proven conflicting. In an initial genome-wide case-control screen involving 65,671 single nucleotide polymorphisms (SNPs) from 13,738 genes in 1,133 MI cases and 1,006 controls in Japan, susceptibility to MI appeared to be associated with the *LTA* A252G polymorphism [16]. Associations between G10A and C804A and susceptibility to MI have subsequently been observed in some studies [17]–[19], but not others [20]–[23]. To further clarify these inconsistent association findings and to identify possible pathogenic polymorphisms in the *LTA* gene in relation to MI, we performed a meta-analysis using published data from observational studies.

## Materials and Methods

### Identification of Relevant Studies

A literature search for GAS that investigated the association between the *LTA* genetic polymorphisms and susceptibility to MI published before October 15, 2013 was conducted in the following electronic databases: PubMed, Embase, Web of Science, Cochrane Library, and CNKI (China National Knowledge Infrastructure) databases. The following combinations of main keywords were used: (‘lymphotoxin-alpha’ or ‘LTA’ or ‘tumor necrosis factor beta’ or ‘TNF-beta’) and (‘myocardial infarction’ or ‘myocardial infarct’ or ‘MI’) and (‘genetic polymorphism’ or ‘single nucleotide polymorphisms’ or ‘SNP’). The search was done without limitations on language but only included those studies that were conducted on human subjects. All references in eligible articles were extensively reviewed to identify additional published articles.

### Inclusion and Exclusion Criteria

To be included in the analysis, eligible studies had to meet the following criteria: (1) case-control studies on the association between the *LTA* genetic polymorphisms and susceptibility to MI; (2) all patients in the candidate studies meet the diagnostic criteria for MI; (3) studies with sufficient available data to calculate ORs with corresponding 95%CIs. The major reasons for excluding studies were: (1) not case-control study; (2) duplicate publications with overlapping subjects from the same study; and (3) no available data reported. For multiple studies using overlapping cases or controls, the most recent study with the largest sample size was included in the meta-analysis. This meta-analysis was conducted according to the Preferred Reporting Items for Systematic Reviews and Meta-analyses (PRISMA) guidance with only slight modification, and did not require ethics board approval (Supplement S1) [24].

### Data Extraction

According to the PRISMA guidance, two investigators independently checked each full-text report for eligibility and extracted the following data from eligible studies: surname of first author, year of publication, country of origin, ethnicity, definition and number of case and control, age, sex ratio, genotyping method, allele and genotype frequency, etc. Disagreements were solved by discussion between all authors until consensus was reached. For data not provided in table form or in the main text, required information was obtained by contacting corresponding authors when possible.

### Quality Assessment

The strengthening report of genetic association studies (STREGA) quality score system and the Newcastle-Ottawa Scale (NOS) criteria were used to assess the qualities of all included studies [25], [26]. The STREGA system includes twenty-two quality assessment items with scores ranging from 0 to 22 (Supplement S2). Studies are classified into three levels based on their scores: low quality (0–12), moderate-high quality (13–17), and high quality (18–22). The NOS criteria use a “star” rating system to judge methodological quality based on three aspects of a study: selection, comparability, and exposure (Supplement S3). Scores range from 0 stars (worst) to 9 stars (best), with a score of 5 or higher indicating a moderate-high methodological quality. Two authors independently assessed the quality of included studies. Discrepancies over quality scores were resolved by discussion with all authors and subsequent consensus.

### Statistical Analysis

Genotype distributions in the controls were tested for conformation to Hardy-Weinberg equilibrium (HWE) using the chi-square test. HWE in the controls was tested by comparing the expected and observed genotype frequencies using the Pearson chi-square test for goodness of fit. The association between the *LTA* genetic polymorphisms and susceptibility to MI was assessed by the pooled odds ratios (ORs) with their corresponding 95% confidence intervals (95%CIs) under five genetic models, including the allele model, the dominant model, the recessive model, the homozygous model and the heterozygous model. Taking into consideration possible between-study heterogeneity, a statistical test for heterogeneity was first conducted using Cochran's Q statistic and the *I^2^* metric [27], [28]. We considered the presence of significant heterogeneity at the 10% level of significance and values of *I^2^* exceeding 50% as an indicator of significant heterogeneity. When no heterogeneity was found with *P*>0.10 or *I^2^*<50%, a fixed-effects model was used to estimate the pooled ORs and 95%CIs. Otherwise, a random-effects model was applied. In addition to an overall comparison, stratified analyses, based on ethnicity, source of control, HWE status, and genotyping method where applicable, were also performed to explore possible explanations of between-study heterogeneity and to investigate whether overall reported associations were present in subgroups. Univariate and multivariate meta-analyses were conducted to explore potential sources of heterogeneity, considering publication year, ethnicity, genotyping method, source of control, and quality score. Sensitivity analyses were conducted by omitting individual studies in turn to reflect the influence of individual datasets on the pooled results [29]. Begg's funnel plot and Egger's linear regression test were used to assess the potential for publication bias [30], [31]. All two-tailed *P*<0.05 were considered statistically significant, and all analyses were performed using the STATA 12.0 software (Stata Corp., College Station, TX, USA).

## Results

### Baseline Characteristics of Included Studies

**Figure 2:**
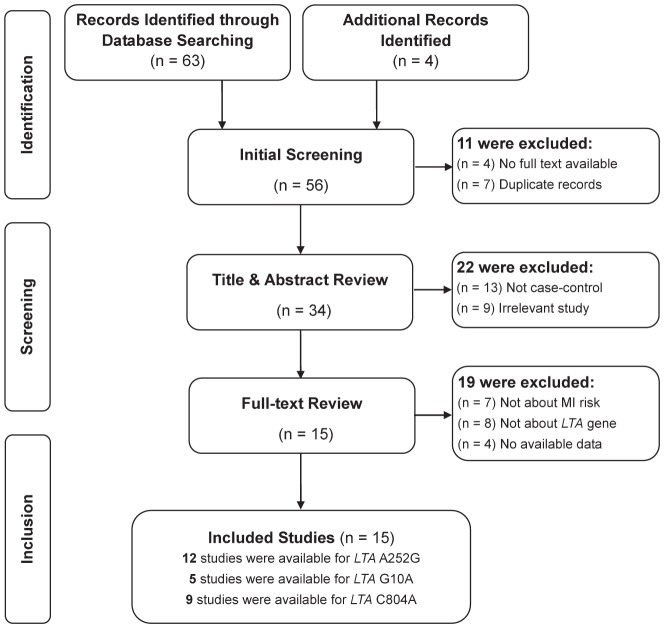
Flow diagram of the selection of studies and specific reasons for exclusion from the present meta-analysis.

Figure 2 presents a flow chart of retrieved and excluded studies with their reasons for exclusion. A total of 67 relevant papers were identified using the pre-specified search strategy. In accordance with the inclusion criteria, fifteen case-control studies [14], [16]–[23], [32]–[37] were included in this meta-analysis, with a total of 22,549 MI patients and 16,105 healthy controls. Of the included studies, twelve examined associations with A252G (one study reported data separately for Japanese and Korean), five examined associations with G10A, and nine examined associations with C804A. Studies were conducted in two major ethnic populations, with seven on Asians and eight on Caucasians. The publication years of included studies ranged from 2000 to 2010. All studies in this meta-analysis had controls in HWE, except one study conducted by Ozaki et al., which was excluded in further stratified analyses. The quality scores of all included studies were moderate-high, with STREGA scores higher than 13 and NOS stars more than 5. The characteristics and methodological quality of all included studies are summarized in Table 1.

**Table 1:** Main characteristics and methodological quality of all eligible studies.

First author, year	Country	Ethnicity	No. of cases, age(y)	No. of controls, age(y)	Male (%) [Case/Control]	Genotyping method	Variant(s)	STREGA score	NOS star
Padovani JC, 2000	Brazil	Caucasian	148, mean 43 range (25–55)	148, mean 42 range (22–55)	82.5%, 82.5%	PCR-RFLP	A252G	19/22	8/9
Koch W, 2001	Germany	Caucasian	793, mean 62.6 (SD 10.2)	340, mean 63.4 (SD 10.3)	77.4%, 75.3%	AS-PCR	A252G	17/22	7/9
Iwanaga Y, 2004	Japan	Asian	477, mean 56 (SD 8)	372, mean 59 (SD 9)	100%, 100%	TaqMan	A252G, G10A, C804A	18/22	7/9
Tobin MD, 2004	UK	Caucasian	547, mean 61.9 (SD 9.2)	505, mean 58.6 (SD 10.7)	68%, 62%	PCR-RFLP	C804A	16/22	6/9
Yamada A, 2004	Japan	Asian	1891, mean 60.6 (SD 10.9)	1798, mean 58.6 (SD 11.3)	78.9%, 55.2%	AS-PCR	A252G, C804A	17/22	7/9
Ozaki K, 2005	Japan	Asian	1133, mean 62.5 (SD 11.3)	1006, mean 64.3 (SD 11.3)	NA, NA	PCR-RFLP	A252G, G10A, C804A	20/22	8/9
Clarke R, 2006	UK	Caucasian	6928, mean 54.8 (SD 7.3)	2712, mean 46.2 (SD 9.6)	82.3%, 44.6%	TaqMan	A252G, G10A, C804A	19/22	8/9
Tanaka T, 2006	Japan	Asian	2833, NA	3399, NA	NA, NA	PCR-RFLP	C804A	15/22	6/9
Kimura A, 2007	Japan	Asian	533, mean 59.4 (SD 10.1)	683, mean 43.3 (SD 10.9)	83.3%, 67.7%	PCR-RFLP	A252G	17/22	7/9
Kimura A, 2007	Korea	Asian	448, mean 61.3 (SD 11.9)	160, mean 61.3 (SD 8.8)	82.4%, 83.5%	PCR-RFLP	A252G	17/22	7/9
Koch W, 2007	Germany	Caucasian	3657, mean 64.0 (SD 12.0)	1211, mean 60.3 (SD 11.9)	75.8%, 50.0%	TaqMan	A252G, G10A, C804A	19/22	8/9
Sedlacek K, 2007	Germany	Caucasian	1821, mean 58.7 (SD 8.6)	2572, mean 57.4 (SD 9.7)	78.0%, 43.2%	TaqMan	G10A, C804A	18/22	7/9
Panoulas VF, 2008	UK	Caucasian	388, mean 61.5 (SD 12.0)	399, mean 50.2 (SD 15.8)	26.8%, 39.6%	PCR-MCA	A252G	16/22	6/9
Ryan AW, 2008	Irish	Caucasian	835, mean 61.9 (SD 8.5)	691, mean 52.4 (SD 10.0)	NA, NA	PCR-RFLP	A252G	15/22	6/9
Wang YC, 2010	Taiwan	Asian	117, NA	109, NA	NA, NA	AS-PCR	A252G, C804A	14/22	5/9

NA, Not available; SD, Standard deviate; PCR-RFLP, Polymerase chain reaction-restriction fragment length polymorphism; AS-PCR, Allele specific-polymerase chain reaction; PCR-MCA, Polymerase chain reaction-melting curve analysis; STREGA, Strengthening the reporting of genetic association studies; NOS, Newcastle-Ottawa scale.

### Association between the *LTA* A252G Polymorphism and Susceptibility to MI

**Figure 3:**
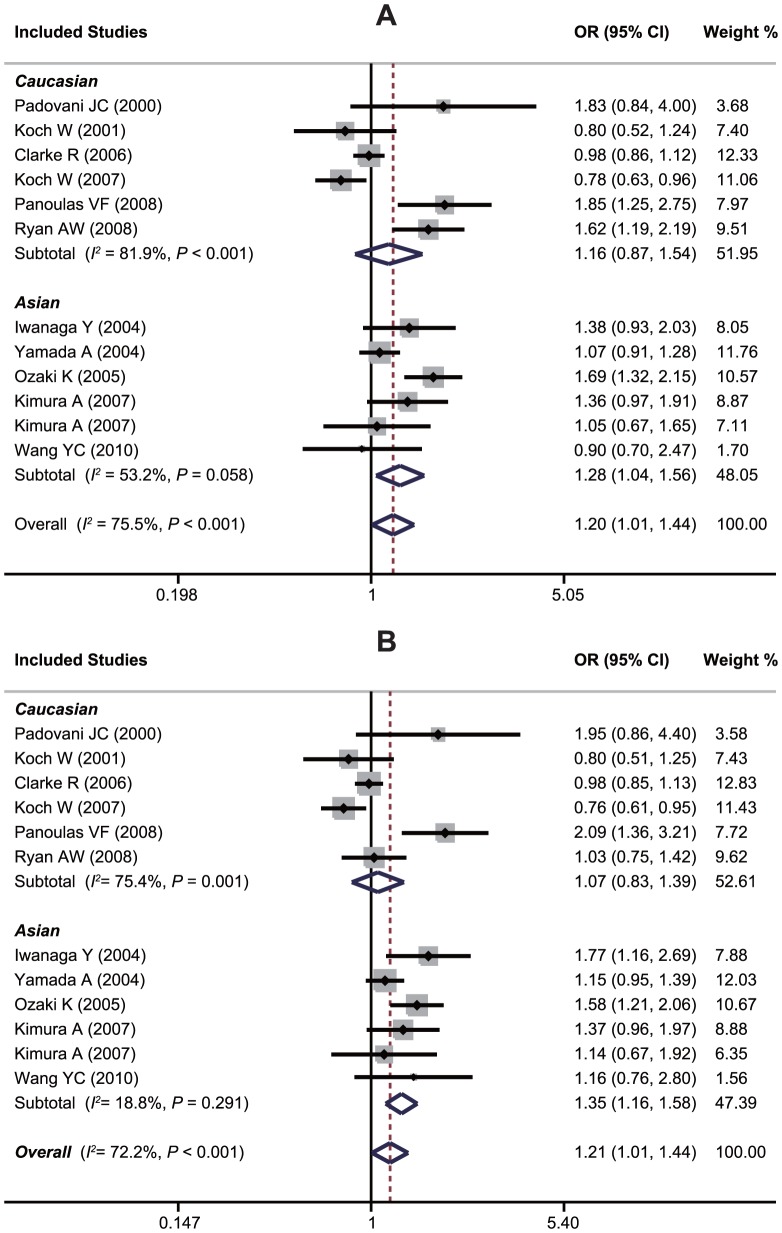
Forest plots of ORs for the association between the *LTA* A252G polymorphism and susceptibility to myocardial infarction in subgroup analysis based on ethnicity under the recessive model (A) and the homozygous model (B).

An evaluation of the association between the *LTA* A252G polymorphism and susceptibility to MI is summarized in Table 2. Twelve case-control studies investigated the relationship between A252G and susceptibility to MI with a total of 17,348 MI patients and 9,629 healthy controls. Since between-study heterogeneity obviously existed (*P*<0.10 and *I^2^*>50% under all genetic models), the random-effects model was used. As shown in Table 2, a borderline significant overall association was found, which indicates that GG genotype may confer an increased susceptibility to MI compared with AA and AG genotypes under recessive and homozygous genetic models (GG vs. AA+AG: OR = 1.20, 95%CI: 1.01–1.44, *P* = 0.040; GG vs. AA: OR = 1.21, 95%CI: 1.01–1.45, *P* = 0.039). However, exclusion of one hospital-based study by Yamada et al. made the summary ORs in population-based studies become insignificant, and after excluding of one non-HWE study by Ozaki et al., the pooled ORs in HWE studies also became insignificant, suggesting that the A252G polymorphism is unlikely to have a major role in the risk of MI. Interestingly, in a stratified analysis by ethnicity, we found that this polymorphism played different roles in Asian and Caucasian populations. In Asians, subjects harboring the 252G variant are approximately 20% more likely to have a MI when compared to subjects with the 252A allele (G allele vs. A allele: OR = 1.16, 95%CI: 1.07–1.26, *P*<0.001; AG+GG vs. AA: OR = 1.26, 95%CI: 1.04–1.54, *P* = 0.020; GG vs. AA+AG: OR = 1.28, 95%CI: 1.04–1.56, *P* = 0.018; GG vs. AA: OR = 1.35, 95%CI: 1.16–1.58, *P*<0.001). However, no significant effects were found for the Caucasians (all *P*>0.05) (Figure 3). Stratification based on genotyping method showed significant associations between *LTA* A252G and susceptibility to MI in the PCR-RFLP subgroup (GG vs. AA+AG: OR = 1.52, 95%CI: 1.30–1.77, *P*<0.001; GG vs. AA: OR = 1.33, 95%CI: 1.09–1.62, *P* = 0.005; GG vs. AA: OR = 1.69, 95%CI: 1.16–1.58, *P*<0.001), whereas no significant association was found in the non-PCR-RFLP subgroup (all *P*>0.05). Univariate and multivariate meta-analyses further confirmed the above results of subgroup analysis, which suggested that ethnicity might be the major source of between-study heterogeneity with 87% was explained by this covariate (Supplement S4).

**Table 2:** Meta-analysis of the association between *LTA* A252G and the risk of myocardial infarction (MI).

Subgroups	No. of study	G allele vs. A allele			AG+GG vs. AA			GG vs. AA+AG			GG vs. AA			GG vs. AG		
	(Case/Control)	OR	95%CI	P	OR	95%CI	P	OR	95%CI	P	OR	95%CI	P	OR	95%CI	P
Overall	12 (17,348/9,629)	1.08	0.99–1.18	0.082	1.08	0.93–1.26	0.306	1.20	1.01–1.44	0.040	1.21	1.01–1.45	0.039	1.23	0.98–1.55	0.069
Ethnicity																
Caucasian	6 (12,749/5,501)	1.00	0.88–1.13	0.970	0.93	0.75–1.16	0.534	1.16	0.88–1.54	0.303	1.07	0.83–1.39	0.607	1.30	0.87–1.94	0.201
Asian	6 (4,599/4,128)	1.16	1.07–1.26	<0.001	1.26	1.04–1.54	0.020	1.28	1.04–1.56	0.018	1.35	1.16–1.58	<0.001	1.21	0.94–1.55	0.142
Source of control																
PB	11 (15,457/7,831)	1.09	0.98–1.20	0.113	1.09	0.91–1.29	0.358	1.22	0.99–1.51	0.056	1.23	0.99–1.51	0.058	1.26	0.97–1.64	0.088
HB	1 (1,891/1,798)	1.08	0.98–1.19	0.103	1.13	0.98–1.29	0.083	1.08	0.91–1.28	0.409	1.15	0.95–1.39	0.155	1.03	0.86–1.23	0.789
HWE status																
HWE	11 (16,215/8,623)	1.08	0.98–1.18	0.141	1.10	0.92–1.30	0.295	1.15	0.97–1.36	0.105	1.16	0.97–1.40	0.100	1.18	0.94–1.49	0.149
Non-HWE	1 (1,133/1,006)	1.16	1.03–1.31	0.019	1.02	0.85–1.22	0.838	1.69	1.32–2.15	<0.001	1.58	1.21–2.06	0.001	1.77	1.37–2.29	<0.001
Genotyping method																
PCR-RFLP	5 (3,097/2,688)	1.06	0.90–1.25	0.506	0.93	0.66–1.32	0.699	1.52	1.30–1.77	<0.001	1.33	1.09–1.62	0.005	1.69	1.16–2.44	0.006
Non-PCR-RFLP	7 (14,251/6,941)	1.10	0.98–1.23	0.105	1.18	1.00–1.40	0.049	1.05	0.87–1.27	0.639	1.14	0.90–1.45	0.289	0.99	0.85–1.16	0.915

OR, Odd ratio; 95%CI, 95% confidence interval; HB, Hospital-based; PB, Population-based; HWE, Hardy-Weinberg equilibrium; PCR-RFLP, Polymerase chain reaction-restriction fragment length polymorphism.

### Association between the *LTA* G10A Polymorphism and Susceptibility to MI

**Figure 4:**
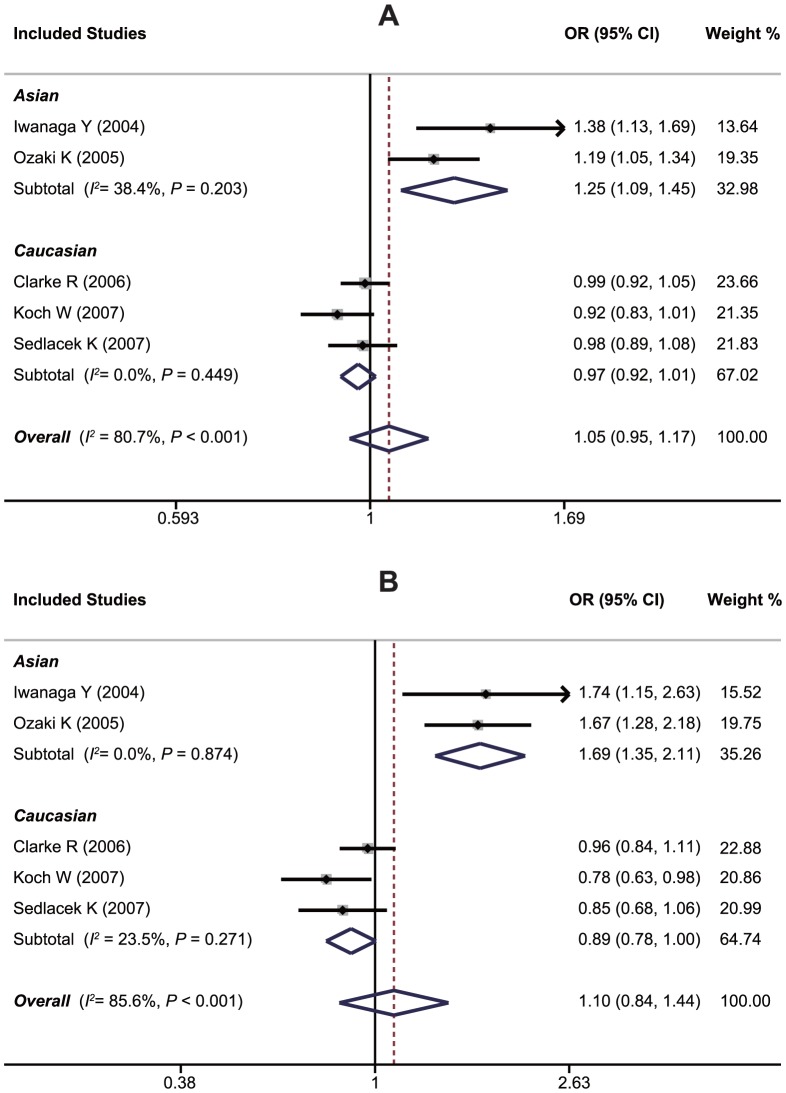
Forest plots of ORs for the association between the *LTA* G10A polymorphism and susceptibility to myocardial infarction in subgroup analysis based on ethnicity under the allele model (A) and the homozygous model (B).

A summary of findings on the relationship between the *LTA* G10A polymorphism and susceptibility to MI is provided in Table 3. Data from five studies, in total compromised of 14,653 MI cases and 7,873 healthy controls, were pooled together for analysis. The random-effects model was conducted since heterogeneity obviously existed (*P*<0.10 and *I^2^*>50% under all genetic models). The overall analysis showed that *LTA* G10A may not be associated with the risk of MI under any of the five genetic models (A allele vs. G allele: OR = 1.05, 95%CI: 0.95–1.17, *P* = 0.349; GA+AA vs. GG: OR = 1.01, 95%CI: 0.96–1.07, *P* = 0.651; AA vs. GG+GA: OR = 1.07, 95%CI: 0.82–1.41, *P* = 0.618; AA vs. GG: OR = 1.10, 95%CI: 0.84–1.44, *P* = 0.498; AA vs. GA: OR = 1.00, 95%CI: 0.91–1.10, *P* = 0.975). Exclusion of one non-HWE study by Ozaki et al. did not make the summary ORs significant in HWE studies. However, stratified analyses based on ethnicity suggested that the 10A allele may confer a significantly increased susceptibility to MI in Asians (A allele vs. G allele: OR = 1.25, 95%CI: 1.09–1.45, *P* = 0.002; GA + AA vs. GG: OR = 1.17, 95%CI: 1.01–1.36, *P* = 0.034; AA vs. GG + GA: OR = 1.63, 95%CI: 1.27–2.08, *P*<0.001; AA vs. GG: OR = 1.69, 95%CI: 1.35–2.11, *P*<0.001; AA vs. GA: OR = 1.61, 95%CI: 1.30–2.00, *P*<0.001), but not in Caucasians, as shown in Figure 4. Due to insufficient observations, meta-regression analyses were not conducted for this SNP.

**Table 3:** Meta-analysis of the association between *LTA* G10A and myocardial infarction risk.

Genetic model	Subgroup	No. of study (CA/CO)	OR [95% CI]	P_OR_	P_h_	Method
A allele vs. G allele	Overall	5 (14,653/7,873)	1.05 [0.95, 1.17]	0.349	<0.001	RE
(Allele model)	Asian	2 (1,610/1,378)	1.25 [1.09, 1.45]	0.002	0.203	FE
	Caucasian	3 (12,406/6,495)	0.97 [0.92, 1.02]	0.179	0.449	FE
	HWE	4 (12,883/6,867)	1.02 [0.92, 1.13]	0.749	0.004	RE
	Non-HWE	1 (1,133/1,006)	1.19 [1.05, 1.34]	0.007	-	FE
GA + AA vs. GG	Overall	5 (14,653/7,873)	1.01 [0.96, 1.07]	0.651	0.014	RE
(Dominant model)	Asian	2 (1,610/1,378)	1.17 [1.01, 1.36]	0.034	0.010	RE
	Caucasian	3 (12,406/6,495)	0.99 [0.93, 1.05]	0.682	0.520	FE
	HWE	4 (12,883/6,867)	1.01 [0.95, 1.08]	0.737	0.006	RE
	Non-HWE	1 (1,133/1,006)	1.04 [0.87, 1.24]	0.682	-	FE
AA vs. GG + GA	Overall	5 (14,653/7,873)	1.07 [0.82, 1.41]	0.618	<0.001	RE
(Recessive model)	Asian	2 (1,610/1,378)	1.63 [1.27, 2.08]	<0.001	0.255	FE
	Caucasian	3 (12,406/6,495)	0.88 [0.78, 1.00]	0.051	0.222	FE
	HWE	4 (12,883/6,867)	0.92 [0.78, 1.09]	0.350	0.062	RE
	Non-HWE	1 (1,133/1,006)	1.78 [1.39, 2.27]	<0.001	-	FE
AA vs. GG	Overall	5 (14,653/7,873)	1.10 [0.84, 1.44]	0.498	<0.001	RE
(Homozygous model)	Asian	2 (1,610/1,378)	1.69 [1.35, 2.11]	<0.001	0.874	FE
	Caucasian	3 (12,406/6,495)	0.89 [0.78, 1.00]	0.059	0.271	FE
	HWE	4 (12,883/6,867)	0.97 [0.78, 1.22]	0.799	0.008	RE
	Non-HWE	1 (1,133/1,006)	1.67 [1.28, 2.18]	<0.001	-	FE
AA vs. GA	Overall	5 (14,653/7,873)	1.00 [0.91, 1.10]	0.975	<0.001	RE
(Heterozygous model)	Asian	2 (1,610/1,378)	1.61 [1.30, 2.00]	<0.001	0.035	RE
	Caucasian	3 (12,406/6,495)	0.89 [0.80, 0.99]	0.030	0.219	FE
	HWE	4 (12,883/6,867)	0.90 [0.79, 1.02]	0.087	0.252	FE
	Non-HWE	1 (1,133/1,006)	1.87 [1.44, 2.41]	<0.001	-	FE

CA, Case group; CO, Control group; OR, Odd ratio; 95%CI, 95% confidence interval; *P_h_*, P value of heterogeneity; HWE, Hardy-Weinberg equilibrium; RE, Random-effect model; FE, Fix-effect model.

### Association between the *LTA* C804A Polymorphism and Susceptibility to MI

**Figure 5:**
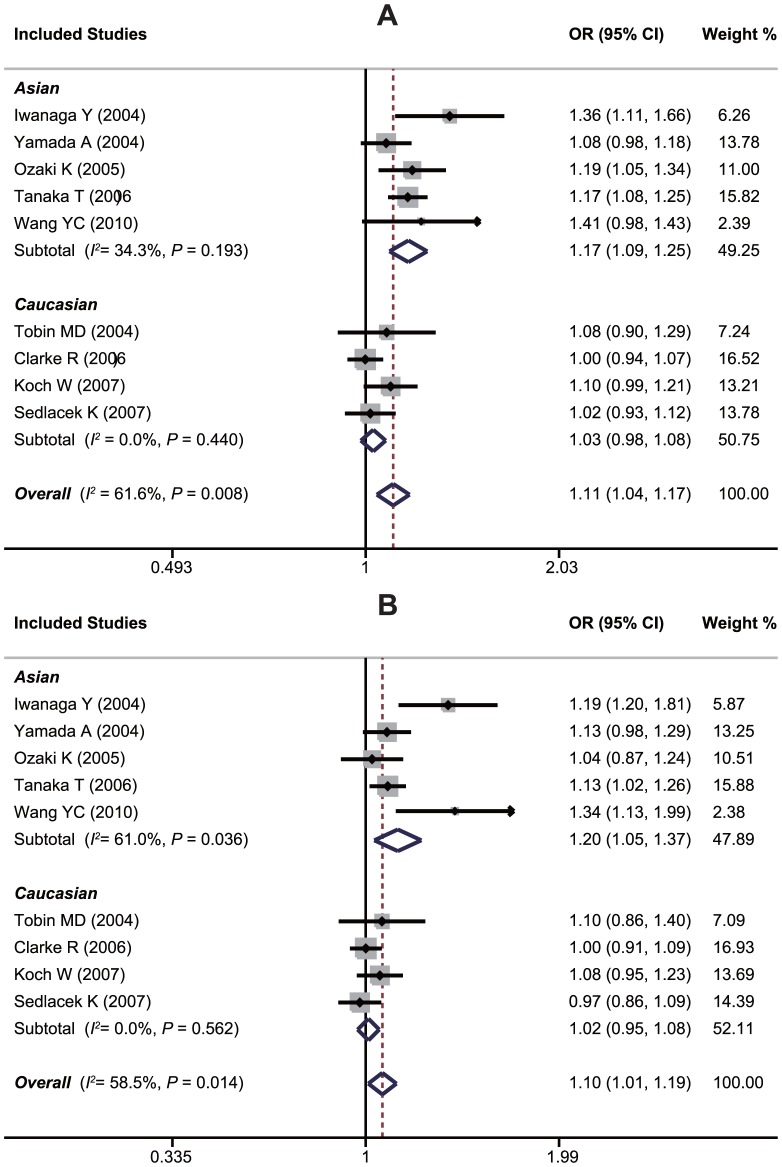
Forest plots of ORs for the association between the *LTA* C804A polymorphism and susceptibility to myocardial infarction in subgroup analysis based on ethnicity under the allele model (A) and the dominant model (B).

Table 4 summarizes the association between the *LTA* C804A polymorphism and susceptibility to MI. Nine case-control studies had investigated the relationship between C804A and susceptibility to MI with a total of 19,404 MI patients and 13,684 healthy controls. Since between-study heterogeneity obviously existed (*P*<0.10 and *I^2^*>50% under all genetic models), the random-effects model was used. Overall, the *LTA* C804A polymorphism was found to be associated with a significant increased susceptibility to MI (A allele vs. C allele: OR = 1.11, 95%CI: 1.04–1.17, *P* = 0.001; CA+AA vs. CC: OR = 1.10, 95%CI: 1.01–1.19, *P* = 0.020; AA vs. CC+CA: OR = 1.24, 95%CI: 1.08–1.41, *P* = 0.002; AA vs. CC: OR = 1.21, 95%CI: 1.01–1.45, *P*<0.001; AA vs. CA: OR = 1.21, 95%CI: 1.04–1.39, *P* = 0.012). As shown in Table 4, neither the exclusion of one hospital-based study by Yamada et al. nor of one non-HWE study by Ozaki et al. changed the summary ORs significantly in population-based studies and HWE studies. Like the other two variants of the *LTA* gene involved in the current study, stratified analysis based on ethnicity revealed a significant association in Asians, but not in Caucasians (Figure 5). Stratification based on genotyping method showed a significant association between *LTA* C804A and susceptibility to MI in the PCR-RFLP subgroup (A allele vs. C allele: OR = 1.16, 95%CI: 1.10–1.23, *P*<0.001; CA+AA vs. CC: OR = 1.11, 95%CI: 1.02–1.20, *P* = 0.018; AA vs. CC+CA: OR = 1.45, 95%CI: 1.17–1.79, *P* = 0.001; AA vs. CC: OR = 1.46, 95%CI: 1.26–1.69, *P*<0.001; AA vs. CA: OR = 1.44, 95%CI: 1.11–1.86, *P* = 0.006), whereas no significant association was found in the other subgroups (all *P*>0.05). Similar to A252G, univariate and multivariate meta-analyses also suggested that ethnicity might be the major source of between-study heterogeneity for C804A with 83% was explained by this covariate (Supplement S4).

**Table 4:** Meta-analysis of the association between *LTA* C804A and the risk of myocardial infarction (MI).

Subgroups	No. of study	A allele vs. C allele			CA+AA vs. CC			AA vs. CC+CA			AA vs. CC			AA vs. CA		
	(Case/Control)	OR	95%CI	P	OR	95%CI	P	OR	95%CI	P	OR	95%CI	P	OR	95%CI	P
Overall	9 (19,404/13,684)	1.11	1.04–1.17	0.001	1.10	1.01–1.19	0.020	1.24	1.08–1.41	0.002	1.21	1.01–1.45	<0.001	1.21	1.04–1.39	0.012
Ethnicity																
Caucasian	4 (12,953/7,000)	1.03	0.98–1.08	0.234	1.02	0.95–1.08	0.628	1.11	0.98–1.26	0.097	1.27	1.11–1.45	0.099	1.11	0.98–1.26	0.113
Asian	5 (6,451/6,684)	1.17	1.09–1.25	<0.001	1.20	1.05 –1.37	0.007	1.35	1.10–1.66	0.004	1.41	1.20–1.66	<0.001	1.28	1.00–1.65	0.052
Source of control																
PB	8 (17,513/11,886)	1.11	1.04–1.19	0.003	1.10	1.00–1.20	0.043	1.27	1.09–1.48	0.002	1.29	1.11–1.51	0.001	1.24	1.06–1.46	0.009
HB	1 (1,891/1,798)	1.08	0.98–1.18	0.113	1.13	0.98–1.29	0.083	1.07	0.90–1.27	0.459	1.14	0.94–1.38	0.174	1.02	0.85–1.22	0.857
HWE status																
HWE	8 (18,271/12,678)	1.10	1.03–1.17	0.005	1.11	1.02–1.21	0.021	1.18	1.05–1.32	0.006	1.22	1.07–1.39	0.002	1.14	1.02–1.28	0.024
Non-HWE	1 (1,133/1,006)	1.19	1.05–1.34	0.007	1.04	0.87–1.24	0.689	1.78	1.39–2.27	<0.001	1.67	1.28–2.18	<0.001	1.87	1.44–2.41	<0.001
Genotyping method																
PCR-RFLP	3 (4,513/4,910)	1.16	1.10–1.23	<0.001	1.11	1.02–1.20	0.018	1.45	1.17–1.79	0.001	1.46	1.26–1.69	<0.001	1.44	1.11–1.86	0.006
TaqMan	4 (12,883/6,867)	1.07	0.98–1.18	0.132	1.08	0.95–1.23	0.255	1.14	0.99–1.30	0.072	1.18	0.99–1.42	0.069	1.11	0.97–1.26	0.120
AS-PCR	2 (2,008/1,907)	1.16	0.92–1.47	0.215	1.36	0.85–2.16	0.196	1.07	0.90–1.26	0.455	1.15	0.95–1.39	0.151	1.01	0.85–1.21	0.891

OR, Odd ratio; 95%CI, 95% confidence interval; HB, Hospital-based; PB, Population-based; HWE, Hardy-Weinberg equilibrium; PCR-RFLP, Polymerase chain reaction-restriction fragment length polymorphism.

### Sensitivity Analyses and Publication Bias

**Figure 6:**
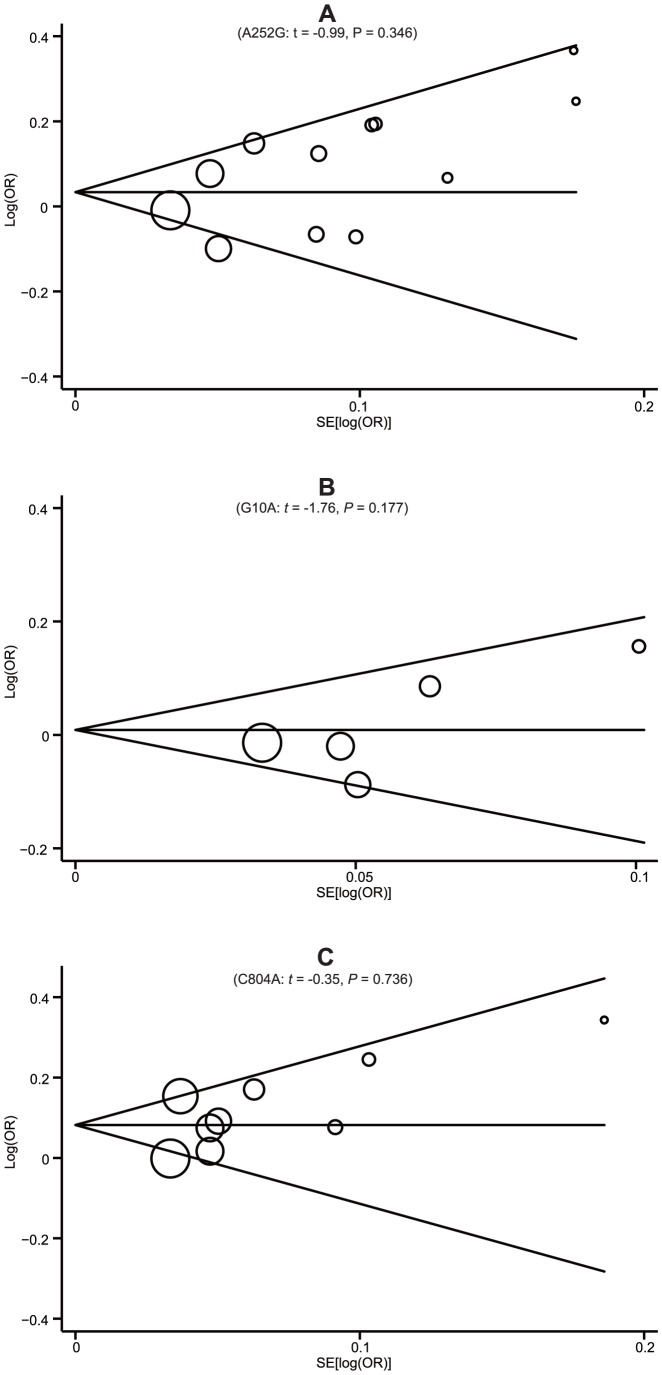
Begg's funnel plots of publication bias for the associations the *LTA* A252G (A), G10A (B) and C804A (C) polymorphisms and susceptibility to myocardial infarction under the allele model.

Sensitivity analyses for *LTA* A252G, G10A and C804A were conducted to determine the influence of individual datasets on the pooled ORs by sequential removal of each eligible study. Omission of one study at a time reveals that the pooled estimates remain virtually the same with each study excluded, indicating that no single study heavily influenced summary ORs in this meta-analysis (data not shown). Begg's funnel plots and Egger's linear regression test were used to assess the potential publication bias of included studies under the allele model. The shapes of the funnel plots did not reveal any evidence of obvious asymmetry (Figure 6). In addition, Egger's test did not show any statistical evidence of publication bias (A252G: *t* = −0.99, *P* = 0.346; G10A: *t* = −1.76, *P* = 0.177; C804A: *t* = −0.35, *P* = 0.736). The above tests indicated a promising level of robustness and accuracy for the results of this meta-analysis.

## Discussion

MI is a multifactorial disease significantly associated with certain genetic factors [38]. Many genome association and candidate gene studies have been conducted to identify MI-susceptible genes, discover novel molecular pathways and help susceptible individuals prevent the development of MI [16], [39]–[41]. To date, several genetic association studies (GAS) have found that SNPs in the *LTA* gene and the LTA protein are associated with MI onset. LTA is a proinflammatory cytokine with homology to inflammatory cytokine tumor necrosis factor alpha (TNF-α) and is related with the development of atherosclerotic lesions in coronary arteries [42]. Common SNPs of the *LTA* gene can modify the function of the LTA protein qualitatively and quantitatively, thereby conferring a risk for MI, as shown in Figure 1. Amino-acid substitutions in the *LTA* gene, such as A-to-C in intron 1, G-to-A in exon 1, and C-to-A in exon 3, may influence the inflammatory biological activities through causing functional mutations in LTA protein, which in turn affect the degree of inflammation and confer a higher susceptibility to MI than normal LTA protein. Based on this fact, several investigations have been conducted to assess the association between common *LTA* genetic polymorphisms and susceptibility to MI. Previously, Padovani et al. first investigated the association between *LTA* A252G and susceptibility to MI in a Brazilian population, but failed to find a significant association [32]. Koch et al. conducted a similar study in a Germany population, and again found no significant results [33]. However, in subsequent studies on Asians, both Iwanaga et al. and Ozaki et al. reported strong evidence for the influence of *LTA* genetic polymorphisms on susceptibility to MI in Japanese populations [34], [37]. Furthermore, Wang et al. observed a significant effect of *LTA* A252G and C804A on susceptibility to MI in a Taiwanese population [19], whereas Kimura et al. did not find any effect in a Korean population [20]. The discrepancy of these studies is partly due to their limited sample sizes and insufficient statistical power to demonstrate significant associations. In addition, these studies included different populations and sampling strategies, making their results difficult to interpret. Thus, in the present study, we performed a meta-analysis to derive a relatively comprehensive assessment of the relationships between the *LTA* A252G, G10A and C804A polymorphisms and susceptibility to MI.

Meta-analysis has the advantage of synthesizing data from published GAS to obtain greater statistical power for detecting significant associations than available in an individual GAS, especially in the absence of large heterogeneity between studies [43]. A large number of meta-analyses have been conducted to investigate the association between the *LTA* gene and various diseases, including gastric cancer [44], [45], breast cancer [46], asthma [47], sepsis [48], migraine [49], and lymphoma [50]. To the best of our knowledge, our study is the first meta-analysis to describe the associations of the *LTA* genetic polymorphisms with susceptibility to MI. This systematic review provides a more comprehensive summary of the currently available evidence on the associations between the *LTA* A252G, G10A and C804A polymorphisms and susceptibility to MI. In this meta-analysis, the *LTA* C804A polymorphisms seem to be associated with an increased susceptibility to MI, whereas *LTA* A252G and G10A may confer a significant increased susceptibility to MI only in Asians.

Heterogeneity is a major problem when interpreting the results of meta-analyses. As the results of meta-regression shows, ethnicity was an important source of this heterogeneity as individuals of different ethnicities may have diverse genetic backgrounds and environmental factors, and as a result the same polymorphism may play different roles in different populations. Interestingly, our subgroup analysis by ethnicity showed that the *LTA* genetic polymorphisms played different roles in Asian and Caucasian populations. All these *LTA* genetic polymorphism had significant increased effects on the risk of MI in Asians, whereas no significant effects were found in Caucasians. These conflicting results may be due to the different genetic backgrounds of these populations, subsequently leading to different genetic susceptibility to the same disease. In addition, the source of controls was another factor that contributed to heterogeneity. The genotype distribution in population-based controls may be similar to normal and thus population-based controls could be more reliable than hospital-based controls. This might partially explain why the results of the stratified analysis by the source of the controls showed differences between the two subgroups for *LTA* A252G. Thus, further studies with larger sample size using population-based controls are warranted. Moreover, subgroup analysis based on genotyping method showed a significant association between *LTA* A252G and C804A and susceptibility to MI in the PCR-RFLP subgroup, whereas no significant association was found in the non-PCR-RFLP subgroups. This result may be due to the overlapping effect of ethnicity, since the significant association was also observed in Asians and most included studies on Asian subjects used the PCR-RFLP genotyping method. In addition, this association may also be due to chance especially for C804A, for which only three studies were involved in the PCR-RFLP subgroups. Hence, further investigations are also warranted to assess the effect of different genotyping methods on the significance of the association between *LTA* genetic polymorphisms and susceptibility to MI.

In interpreting the results of this meta-analysis, some specific issues need to be addressed. First, the significant associations found for the *LTA* A252G and G10A polymorphisms should be interpreted in caution. For A252G, exclusion of hospital-based and non-HWE studies made the summary ORs become insignificant, suggesting that this polymorphism is unlikely to have a major role in susceptibility to MI. For G10A, the significant association in Asians should also be treated carefully, since only two studies on Asians were involved and thus statistical power was limited due to the small sample size. Hence, the significant associations of *LTA* A252G and G10A with susceptibility to MI should be interpreted in caution and more large-scale studies are warranted for confirming these relationships. Second, as with other complex traits, susceptibility to MI may be modulated by other genetic markers besides the *LTA* gene. Thus, fully elucidating the pathogenesis of MI would demand an investigation into the association and combined interaction of many gene variants with susceptibility to MI. Third, the included studies only focused on the Asian and Caucasian populations and thus further studies on a wider spectrum of subjects should be carried out to investigate the role of these variants in different ethnicities. Last, this meta-analysis was based on unadjusted ORs and possible effect modifiers, such as sex, age, BMI, diabetes mellitus, and smoking status, may influence the estimates of associations. Unfortunately, the calculation of adjusted pooled ORs and further subgroup analyses based on these factors could not be performed because of limited data. Thus, further well-designed GAS need to focus on exploring these sources of heterogeneity. Despite these limitations, our study is the first comprehensive meta-analysis of all eligible studies on the association between the *LTA* genetic polymorphisms and MI risk.

In summary, the current meta-analysis indicates that the *LTA* A252G and C804A polymorphisms may be associated with an increased risk of MI, whereas *LTA* G10A may confer a significant increased risk of MI only in Asian populations. Thus, these polymorphisms of the *LTA* gene could possibly be used with other genetic markers together to identify individuals at high risk for MI. However, due to the limitations of this study, these results should be interpreted with caution and still require future large-scale studies to confirm their accuracy. Moreover, considering that MI is a complex disease with a multifactorial etiology, the development of MI might be associated with gene-gene and gene-environment interactions, whose effects should be considered in future GAS and subsequent meta-analyses that may provide more conclusive evidence regarding the genetic susceptibility to MI.

## Supporting Information

Supplement S1 **The Preferred Reporting Items for Systematic Reviews and Meta-analyses (PRISMA) Checklist.**
(DOC)

Supplement S2 **The Strengthening the Reporting of Genetic Association Studies (STREGA) quality score systems.**
(DOC)

Supplement S3 **The Newcastle-Ottawa Scale (NOS) criteria.**
(DOC)

Supplement S4 **Univariate and multivariate meta-analyses of potential source of heterogeneity.**
(DOC)
