# Supplementary material for: Polymorphisms of the LTA Gene May Contribute to the Risk of Myocardial Infarction: A Meta-Analysis
Source: PLoS One. 2014 Mar 18;9(3):e92272. doi: 10.1371/journal.pone.0092272 (PMC3958506; doi:10.1371/journal.pone.0092272)
Supplement: Supplement S3 — The Newcastle-Ottawa Scale (NOS) criteria. (DOC) [file pone.0092272.s003.doc]

**Supplement S3. The Newcastle-Ottawa Scale for assessing methodological quality of eligible studies.**

| **Category** | **Item** | **Explanation** | **Star** |
| --- | --- | --- | --- |
| Selection | (1) Is the case definition adequate | A. Yes, with independent validation | ★ |
|  |  | B. Yes, eg record linkage or based on self reports |  |
|  |  | C. No description |  |
|  | (2) Representativeness of the cases | A. Truly representative | ★ |
|  |  | B. Not satisfying requirements, or not stated. |  |
|  | (3) Selection of controls | A. Community controls | ★ |
|  |  | B. Hospital controls |  |
|  |  | C. No description |  |
|  | (4) Definition of Controls | A. No history of related disease | ★ |
|  |  | B. With history of related diseases |  |
|  |  | C. No mention of history of related diseases |  |
| Comparability | (1) Comparability of cases and controls on the basis of the design or analysis | A. Age | ★ |
|  |  | B. Other controlled factors | ★ |
|  |  | C. No description |  |
| Exposure | (1) Diagnostic criteria for pancreatic cancer | A. NCCN guidelines | ★ |
|  |  | B. Only clinical routine examinations |  |
|  |  | C. No description |  |
|  | (2) Follow-up | A. Yes | ★ |
|  |  | B. No |  |
|  |  | C. No description |  |
|  | (3) Completion rates of genotype detection | A. The majority of cases and controls | ★ |
|  |  | B. Less than 80% patients |  |
|  |  | C. No description |  |

Wells GA, Shea B, O'Connell D, Peterson J, Welch V, Losos M, Tugwell P. The Newcastle-Ottawa Scale (NOS) for assessing the quality of nonrandomised studies in meta-analyses.
<http://www.ohri.ca/programs/clinical_epidemiology/oxford.asp>
