# Supplementary material for: Polymorphisms of the LTA Gene May Contribute to the Risk of Myocardial Infarction: A Meta-Analysis
Source: PLoS One. 2014 Mar 18;9(3):e92272. doi: 10.1371/journal.pone.0092272 (PMC3958506; doi:10.1371/journal.pone.0092272)
Supplement: Supplement S4 — Univariate and multivariate meta-analyses of potential source of heterogeneity. (DOC) [file pone.0092272.s004.doc]

**Supplement S4** Univariate and multivariate meta-analyses of potential source of heterogeneity

| **Heterogeneity factors** | ***ERCC2* K751Q** | | | |  | ***ERCC2* C804A** | | | |
| --- | --- | --- | --- | --- | --- | --- | --- | --- | --- |
|  | ***t*** | ***P*** | ***I^2^*** | ***R^2^*** |  | ***t*** | ***P*** | ***I^2^*** | ***R^2^*** |
| ***Publication year*** |  |  |  |  |  |  |  |  |  |
| **Univariate** | -0.31 | 0.766 | 86.60% | 8.99% |  | 0.28 | 0.785 | 65.27% | 12.66% |
| **Multivariate** | -0.72 | 0.504 | 27.24% | 93.18% |  | -0.30 | 0.791 | 9.01% | 84.15% |
| ***Ethnicity*** |  |  |  |  |  |  |  |  |  |
| **Univariate** | 4.04 | 0.027 | 6.95% | 87.01% |  | 2.91 | 0.023 | 20.37% | 82.86% |
| **Multivariate** | 3.71 | 0.034 | 27.24% | 93.18% |  | 3.65 | 0.022 | 9.01% | 84.15% |
| ***Genotype method*** |  |  |  |  |  |  |  |  |  |
| **Univariate** | -0.82 | 0.432 | 87.46% | 4.47% |  | 1.36 | 0.215 | 47.97% | 59.49% |
| **Multivariate** | 0.45 | 0.671 | 27.24% | 93.18% |  | 0.84 | 0.491 | 9.01% | 84.15% |
| ***Source of control*** |  |  |  |  |  |  |  |  |  |
| **Univariate** | -0.04 | 0.967 | 87.25% | 13.51% |  | 0.32 | 0.756 | 66.39% | 35.36% |
| **Multivariate** | -1.19 | 0.287 | 27.24% | 93.18% |  | 1.21 | 0.351 | 9.01% | 84.15% |
| ***STREGA score*** |  |  |  |  |  |  |  |  |  |
| **Univariate** | 0.39 | 0.701 | 87.35% | 8.66% |  | -0.82 | 0.438 | 57.22% | 23.54% |
| **Multivariate** | 1.17 | 0.293 | 27.24% | 93.18% |  | 0.35 | 0.761 | 9.01% | 84.15% |
| ***NOS star*** |  |  |  |  |  |  |  |  |  |
| **Univariate** | 0.16 | 0.879 | 87.51% | 11.39% |  | -1.11 | 0.305 | 54.01% | 10.73% |
| **Multivariate** | 1.17 | 0.295 | 27.24% | 93.18% |  | -0.50 | 0.666 | 9.01% | 84.15% |

*STREGA* strengthening the reporting of genetic association studies, *NOS* Newcastle-Ottawa scale, *SE* standard error, *95%CI* 95% confidence interval.
